# PEGylated TiO_2_ nanoparticles mediated inhibition of cell migration via integrin beta 1

**DOI:** 10.1080/14686996.2018.1444318

**Published:** 2018-03-08

**Authors:** Qingqing Sun, Koki Kanehira, Akiyoshi Taniguchi

**Affiliations:** a Cellular Functional Nanobiomaterials Group, Research Center for Functional Materials, National Institute for Materials Science, Tsukuba, Japan; b Graduate School of Advanced Science and Engineering, Waseda University, Tokyo, Japan; c Biotechnology Group, TOTO Ltd. Research Institute, Chigasaki, Japan

**Keywords:** Cell migration, inhibition, integrin beta 1, pFAK, TiO_2_-PEG NPs, 30 Bio-inspired and biomedical materials, 211 Scaffold / Tissue engineering / Drug delivery

## Abstract

Nanoparticles (NPs) elicit various physiological responses in cellular environment, and the effect of NPs on cell migration is of high interest. In this work, the effects of NPs on cell migration and their possible mechanisms were studied. Here, we showed that after exposure to pegylated titanium dioxide nanoparticles (TiO_2_-PEG NPs, where PEG stands for the polyethylene glycol), NCI-H292 cells exhibited slower migration than control cells. Furthermore, larger NPs inhibited cell migration much stronger than smaller NPs. Following NP exposure, the cells showed decreased expression of integrin beta 1 and phosphorylated focal adhesion kinase (pFAK), and disrupted F-actin structures. We demonstrated that a possible mechanism involved NP-mediated promotion of the lysosomal degradation of integrin beta 1, thus leading to reduced expression of pFAK and cytoskeletal disruption and inhibited cell migration. Therefore, our results showed that inhibition of NCI-H292 cell migration by NPs is mediated through integrin beta 1, which provides useful information for the application of NPs in cancer therapy and related fields.

## Graphical abstract

**Figure:**
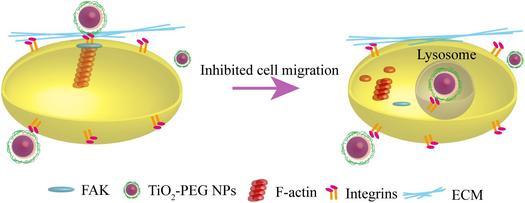

## 1. Introduction

Nanotechnology encompasses numerous, diverse research fields such as biology, engineering, chemistry and physics, among others. Furthermore, nanotechnology research has become integrated into many technological advances with wide applications in scientific, commercial and social fields [1]. Nanomaterials continue to permeate our daily life and environment in the form of industrial, agricultural, medical, cosmetic and food products; a phenomenon that is accompanied by increased risk of exposure to diseases associated with nanomaterials [2–6]. It has been reported that nanomaterials can adversely affect cell viability or physiology [7,8]. Furthermore, more insight is sought on the effects of nanomaterials on cells at the molecular level, such as the generation of reactive oxygen species after cellular uptake [9] and the resultant DNA damage [10,11], apoptosis and cell cycle arrest [12], cell skeletal disruption [13] and other effects. A deeper understanding of nano-bio interactions will provide more reference information for future efforts to develop safe designs for nanotechnology.

Further, there is an immediate need to study the effects of nanomaterials on cell migration, which plays a significant role in determining embryonic development, wound healing, cancer progression, and immune responses [14–17]. Besides that, nanomaterials have been applied for diagnosis and prevention of tumor metastasis [18–20]. Several studies reported the inhibition of cell migration after nanomaterial exposure without clarifying the underlying mechanisms [21,22].

Cell migration is a complex biological process, involving turnover of cell-matrix adhesion sites, the integrin-focal adhesion kinase (FAK) signaling pathway, and the actin cytoskeleton that pulls the cell and directs cell migration. For cell migration, signal transduction from the extracellular environment to the cytoplasm requires integrins as cell surface transducers; they react with the extracellular stimuli, and thus activate the downstream signaling molecule FAK to further propagate the signal to the nuclei, which ultimately controls cell migration [23]. Although many integrins can bind fibronectin, integrin beta 1 is the major fibronectin receptor on most cells [24]. Integrin beta 1 is one of the transmembrane receptors that is activated by extracellular signals. It transmits extracellular signals to the cytoplasm and controls cell adhesion, proliferation and migration, and so on [25,26]. Additionally, integrin beta 1 and nanomaterials are trafficked through the same endocytosis pathways [27].

We hypothesized that integrin beta 1 is involved in nanomaterial endocytosis, and decreases the integrin-FAK signal transduction, thereby inhibiting cell migration. Understanding the molecular mechanisms of how nanomaterials interfere with cell migration will provide important information that will enable nanomaterial designs that lack such adverse effects; it will also improve our general understanding of how nanomaterials affect cell migration.

In this work, the effect of TiO_2_-PEG NPs on cell migration was studied. TiO_2_ NPs are commonly used in foods, cosmetics, toothpaste, medicines, and other commercial products [28–30]. Here, PEG co-polymer was pegylated on the surface of TiO_2_ NPs to increase their hydrodispersity and biocompatibility. Human airway epithelial (NCI-H292) cells, which are derived from human non-small cell lung cancer (NSCLC) and exhibit collective migration, were chosen as a cell model to study the biological interactions between TiO_2_-PEG NPs and cells. We observed a range of phenomena including cell migration, cellular uptake, active integrin beta 1 expression and distribution, elevated FAK activity, and f-actin expression. We found that collective migration of NCI-H292 cells was significantly inhibited in the presence of NPs. This could be attributed to NPs promoting lysosomal degradation of active integrin beta 1 receptor, decreasing expression of active integrin beta 1 and thus weakening the activated FAK signaling and disrupting f-actin structures that inhibit cell migration. Our findings provide a new perspective on how NPs affect cell migration.

## 2. Methods and materials

### 2.1. Cell culture

NCI-H292 cells were cultured in RPMI-1640 culture medium (L-glutamine and Phenol Red) (Nacalai Tesque, Tokyo, Japan) supplemented with 10% (v/v) heat-inactivated fetal bovine serum (FBS) (Biowest, Nuaillé, France), and 100 μg/mL of penicillin and 10 μg/mL of streptomycin (Nacalai Tesque, Tokyo, Japan). Cells were cultured at 37 °C and 5% CO_2_ in 100 mm culture dishes (Wako, Osaka, Japan) and sub-cultured every 2 days.

### 2.2. Preparation of TiO_2_-PEG and FITC-labeled TiO_2_-PEG NPs

TiO_2_-PEG NPs were prepared as previously described [31]. In brief, 0.1 M titanium ethoxide in ethanol was mixed with 50% (v/v) acetonitrile and hydrolyzed for 60 min at room temperature. Ammonium hydroxide was then added to form a hydrolysis solution. The final concentrations of ammonium hydroxide ranged from 0.01 to 0.1% (w/v) and were dependent on the desired particle size. The hydrolysis solution was subsequently heated under reflux. The generated spherical TiO_2_ particles were then collected and finally adjusted to 20% (w/v) with methanol. Finally, a PEG co-polymer was coated on the surface of the spherical TiO_2_ NPs as described previously [32]. The morphology of NPs is displayed in Figure S1 (supplemental information). Besides that, the polydispersity index (PDI) of NPs was detected after dilution and stock in the cell culture medium for 24 h as follows: 0.1 (100 nm), 0.1 (200 nm), and 0.3 (300 nm).

Fluorescein isothiocyanate (FITC, Wako, Osaka, Japan) was mixed with equivalent amounts of dopamine hydrochloride (DA, Wako, Osaka, Japan) in 90% N,N-dimethylformamide/10% N,N-diisopropylethanolamine (v/v) for 1 h at room temperature, and the reaction of the FITC-DA conjugate was monitored by thin layer chromatography on silica gel plates with n-butanol-acetic acid (10/90, v/v) as the mobile phase. For FITC labeling, 1 mM of the FITC-DA conjugate was gently mixed with 0.5 wt% TiO_2_-PEG NPs in an aqueous solution for 24 h at 4 °C. After labeling, the aqueous solution of FITC-labeled TiO_2_-PEG NPs was washed, following centrifugation (20,000 g, 10 min), four times with sterile purified water (more than 99.99% of the solution was replaced) and re-dispersed. A detailed characterization of NPs is provided in our previous work [31].

### 2.3. Cell viability detection

Cell viability assay was carried on as described previously [32]. Firstly, 1 × 10^4^ cells were seeded in a 96-well plate (Corning, NY, USA) and cultured for 24 h. After that, suspension of TiO_2_-PEG NPs with the sizes of 100, 200, and 300 nm were exposed to cells at the concentration of 100 μg/mL. With the exposure to NPs for 3 h, the cell viability was detected using the CellTiter-Glo® luminescent cell viability assay (Promega Corp., Madison, WI, USA) according to the protocol of the kit.

### 2.4. Cell migration measurement

To study the effects of TiO_2_-PEG NPs on cell migration, an *in vitro* scratch assay was performed according to the method described by Liang et al. [33]. NCI-H292 cells were seeded in 6-well plates (Corning, NY, USA) at a density of 8 × 10^4^ cells/cm^2^ and cultured to a 100% confluent monolayer. Prior to seeding, two or three reference lines were created on the back of the 6-well plate using a syringe needle to mark the cell scratch locations for photographing. The scratches on the cell monolayer were then made using p200 pipet tips. A ruler was used to produce straight scratch lines. The distance between the edges of the scratches on the cell monolayer was quickly recorded using an optical microscope. The distances were measured using ImageJ 1.51 (https://imagej.nih.gov/ij/download.html) and set as *D*_0_. The cells were then exposed to the TiO_2_ NPs (100, 200, and 300 nm) at a concentration of 100 μg/mL for 3 h. The distances were then recorded as D_t_. Based on changes in the width of the scratches, the cell migration index was calculated using the following equation [34].

$$\text{Cell migration index}\;\left(\%\right)=\left(D_{0}-D_{t}\right)/D_{0}\times 100\%$$

### 2.5. Quantification of cellular uptake of TiO_2_-PEG NPs

NCI-H292 cells were seeded in 6-well plates at a density of 4 × 10^4^ cells/cm^2^ and cultured overnight to obtain approximately 80% confluence. The cells were then pre-treated with FITC-labeled TiO_2_-PEG NPs (100, 200, and 300 nm) at a concentration of 100 μg/mL for 3 h. The cells were then washed thrice with 1 × phosphate buffered saline (PBS) to completely remove NPs from the cell surface. The cells were then trypsinized, collected and re-suspended in an ice-cold 6% FBS/PBS solution to maintain cellular viability. Finally, the FITC intensity of the NPs was detected using a cell analyzer (SP6800, Sony, Tokyo, Japan) to evaluate differences in cellular uptake.

### 2.6. Flow cytometry assessment of integrin beta 1, pFAK and F-actin

NCI-H292 cells were seeded in 12-well plates (Corning, NY, USA) at a density of 4 × 10^4^ cells/cm^2^ and cultured overnight to allow adhesion. The culture medium was then replaced with fresh culture medium with or without TiO_2_-PEG NPs (100, 200, and 300 nm) at a concentration of 100 μg/mL for 3 h. The cells were then washed thrice with 1 × PBS to completely remove residual NPs, trypsinized and collected by centrifugation at 1200 rpm for 3 min, and finally re-suspended in ice-cold 3% bovine serum albumin (BSA, Wako, Osaka, Japan) solution for subsequent immunostaining. The cells were fixed by replacing the medium with 1% paraformaldehyde (PFA, Wako, Osaka, Japan) for 10 min on ice, followed by replacement with 0.05% Triton X-100 (Wako) for 5 min on ice to permeabilize the cells. The pre-treated cells were then stained for f-actin with rhodamine phalloidin (Cytoskeleton, USA) or primary antibodies at a suitable concentration for at least 30 min on ice. The antibodies used in this study are as follows: phospho-FAK (Tyr397) antibody (31H5L17, Invitrogen, USA) and anti-integrin beta 1 antibody [12G10] (ab30394, Abcam, Cambridge, USA); isotype control: rabbit IgG isotype control (Invitrogen, Waltham, MA, USA) and mouse IgG (ab170190, Abcam, Cambridge, MA, USA). Negative controls were performed using the respective secondary antibodies only. Following primary antibody treatment, the cells were washed and stained with the secondary antibodies (donkey anti-Rabbit IgG (H + L) highly cross-adsorbed secondary antibody (Alexa Fluor 488, Invitrogen, Waltham, MA, USA) and goat anti-mouse IgG/IgM (H + L) secondary antibody (Alexa Fluor 488, Invitrogen) for at least 30 min on ice in the dark. Following washing, the stained cells were re-suspended in 1% BSA solution for flow cytometer analysis (cell analyzer, SP6800, Sony, Tokyo, Japan).

### 2.7. Immunofluorescence observation by confocal microscopy

NCI-H292 cells were seeded in glass bottomed CELLview® cell culture dishes (Greiner Bio-one, Maybachstraße, Frickenhausen, Germany) at a density of 2.5 × 10^4^ cells/cm^2^ and cultured overnight to allow adhesion. The culture medium was replaced with fresh culture medium with or without FITC-TiO_2_-PEG or TiO_2_-PEG NPs for 3 h. The cells were then washed completely and fixed with 4% PFA for 15 min and permeabilized using 0.1% Triton X-100 for 10 min. The cells were subsequently blocked with 3% BSA for 40 min at room temperature and stained with phospho-FAK (Tyr397) antibody, anti-integrin beta 1 antibody [12G10], anti-EEA1 antibody – Early Endosome Marker (ab2900, Abcam), and anti-LAMP1 antibody (ab24170, Abcam) at 4 °C overnight. After washing, cells were stained with the above secondary antibodies and rhodamine phalloidin for 1 h, or 4′,6-diamidino-2-phenylindole (DAPI, Dojin, Yonezawa, Yamagata, Japan) for 5 min, at room temperature. Finally, the stained cells were mounted using anti-fade mounting solution (Fluoromount, Diagnostic Biosystems, Pleasanton, Canada) and observed using a confocal microscope (Leica TCS SP5, Wetzlar, Germany) with an oil 63 × 1.4 plan-apochromat oil-immersion objective.

After confocal observation, the lysosomal integrin beta 1 was quantified. For control, the ratio of lysosomal integrin beta 1 was counted and calculated based on Equation (1). After NPs exposure, to study the effects of NPs on the changes of lysosomal integrin beta 1, the ratio of lysosomal integrin beta 1 was counted and assessed using Equation (2). For each counting, five cells were selected. Besides that, the Pearson correlation coefficient was calculated to display the linear correction between sizes of NPs and lysosomal integrin beta 1.

(1) $$\text{Ratio of lysosomal integrin beta 1 without NPs exposure}=\frac{\text{Numbers of lysosomal integrin beta 1}}{\text{Numbers of lysosomes}}\times 100\%$$

(2) $$\text{Ratio of lysosmal integrin beta 1 with NPs exposure}=\frac{\text{Numbers of lysosomal integrin beta 1 with NPs}}{\text{Numbers of lysosomal NPs}}\times 100\%$$

### 2.8. Statistical analysis

All data were assessed and statistically analyzed using one-way ANOVA. All data are presented as mean ± standard deviation (SD) of at least three parallel groups (*n* ≥ 3). *p ≤ 0.05, **p ≤ 0.01, as shown in the figure legends. *p*-Values ≤0.05 and ≤0.01 indicate significant and highly significant differences.

## 3. Results

### 3.1. TiO_2_-PEG NPs inhibit cell migration in a size dependent manner

**Figure 1.**
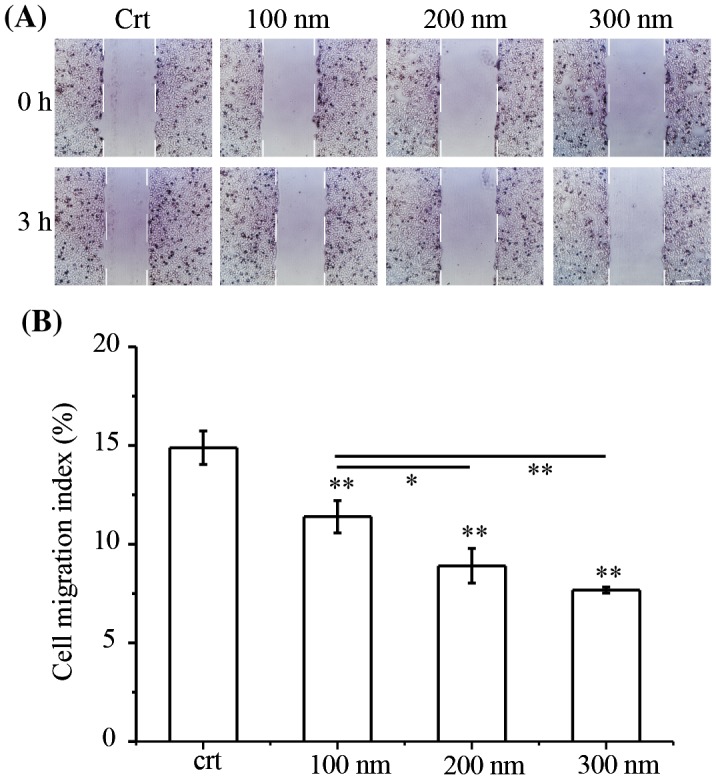
TiO_2_-PEG NPs inhibit cell migration in a size-dependent manner. NCI-H292 cells were seeded overnight prior to the creation of scratches with p200 pipet tips. The cells were then exposed to TiO_2_-PEG NPs (100, 200, and 300 nm) at a concentration of 100 μg/mL for 3 h. (A) Scratch width changes with or without NP exposure, scale bar: 100 μm; (B) Quantification of cell migration with or without NP exposure. Crt stands for control samples. All data are displayed as mean ± SD with at least three parallel groups and were analyzed using one-way ANOVA. *p ≤ 0.05, **p ≤ 0.01.

Determining the effect of NPs on cell migration is vital to understand the biological effects of NPs. It has been reported that NPs adversely affect cell migration [35,36]. However, the molecular mechanism of cell migration inhibition by NPs is not well understood. Therefore, this work examined the effects of various sizes of TiO_2_ NPs on cell migration. Firstly, cell viabilities after exposure to TiO_2_-PEG NPs were checked, founding no cytotoxicity caused by NPs (Figure S2). Following that, cell migration was checked to study the effect of NPs on cell mobility without. Figure 1(A) shows images from the scratch assay, where a monolayer of cells was scratched with p200 pipet tips. The colors in Figure 1(A) were adjusted using Adobe Illustrator 2017 CC software to increase the contrast ratio. After a 3 h incubation in the absence or presence of NPs, the distance between the edges of scratches was measured. The control group (without NPs) showed a narrower scratch width compared to the groups exposed to NPs. In addition, the inhibition of cell migration was proportional to the size of the NPs. To further study the effects of NPs on cell migration, the migration indexes were calculated (Figure 1(B)). Quantification of cell scratch changes confirmed that cell migration is significantly inhibited by NPs. Furthermore, larger NPs had the greatest inhibitory effect on cell migration. Therefore, our results showed that TiO_2_-PEG NPs size-dependently inhibited cell migration.

### 3.2. Expression of integrin beta 1 is decreased by NPs

Exposure to NPs not only causes stress to cell membranes and damages cellular integrity, but also damages the cytoskeleton and cell functions such as cell motility [34,37,38]. Therefore, we determined cellular uptake ratios of TiO_2_-PEG NPs after co-incubation with cells for 3 h. As displayed in Figure 2(A), the cellular uptake ratios of NPs significantly increased in a manner that was proportional to the size of NPs, similar to the observed effect on cell migration. The results suggest that the molecular mechanism of inhibited cell migration by NPs could be related to cellular uptake of NPs.

Figure 2.Integrin beta 1 is transferred from early endosome/macropinosomes to lysosomes with TiO_2_-PEG NPs. Cells were seeded and exposed to the TiO_2_-PEG NPs (100, 200, and 300 nm) at a concentration of 100 μg/mL for 3 h. (A) Quantification of cellular uptake ratios by flow cytometry; (B) Co-localization of integrin beta 1 with FITC-TiO_2_-PEG NPs in early endosomes and macropinosomes; (C) Co-localization of integrin beta 1 with FITC-TiO_2_-PEG NPs in lysosomes; (D) Quantification of changes in integrin beta 1 expression. Crt stands for control samples. All data are displayed as mean ± SD with at least three parallel groups and were analyzed using one-way ANOVA. *p ≤ 0.05, **p ≤ 0.01.

**Figure:**
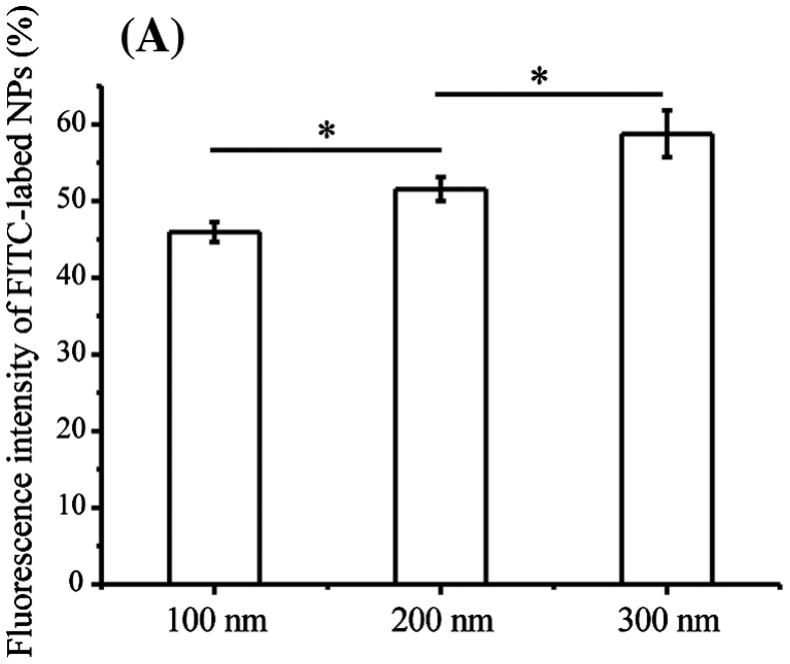

**Figure:**
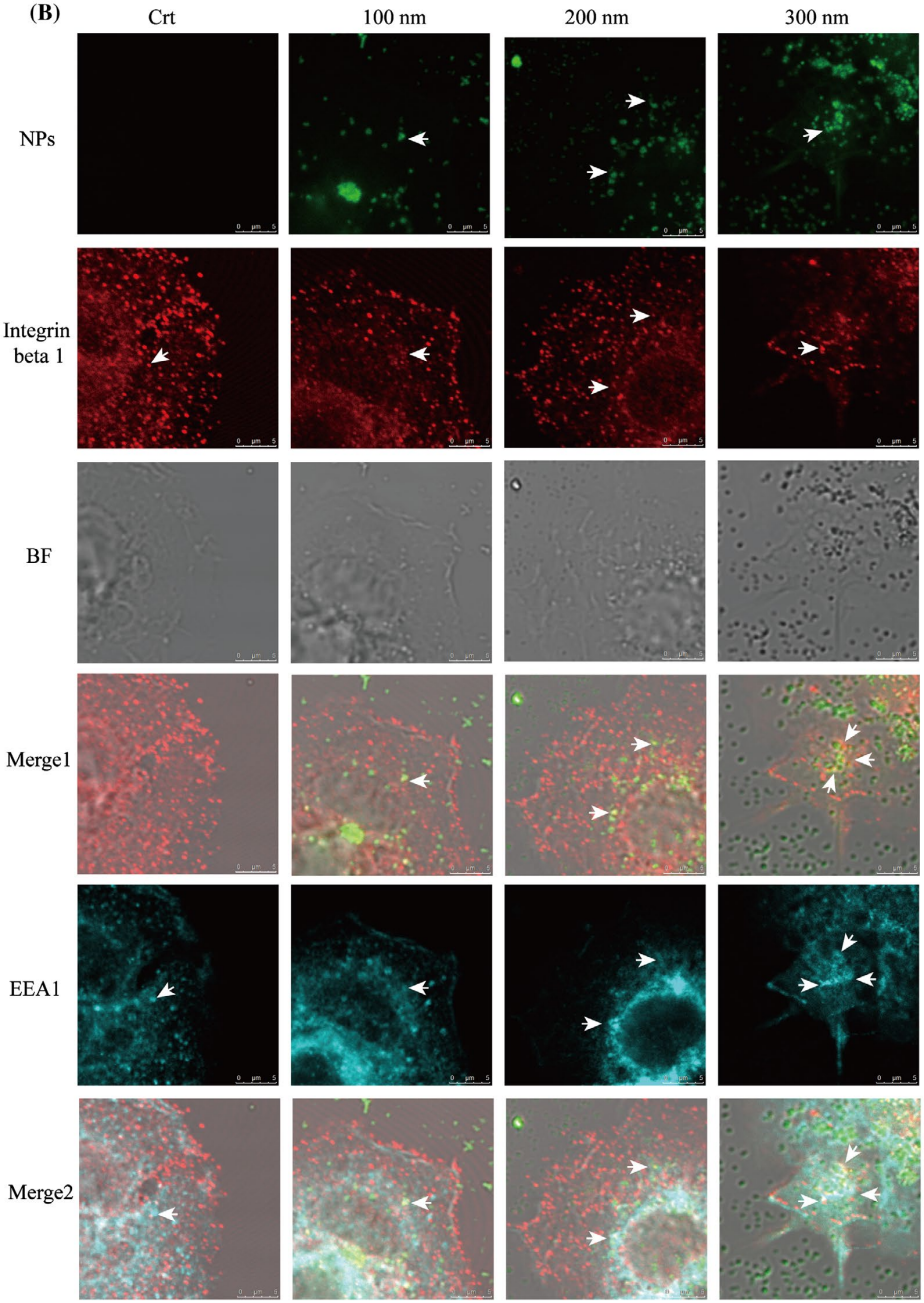

**Figure:**
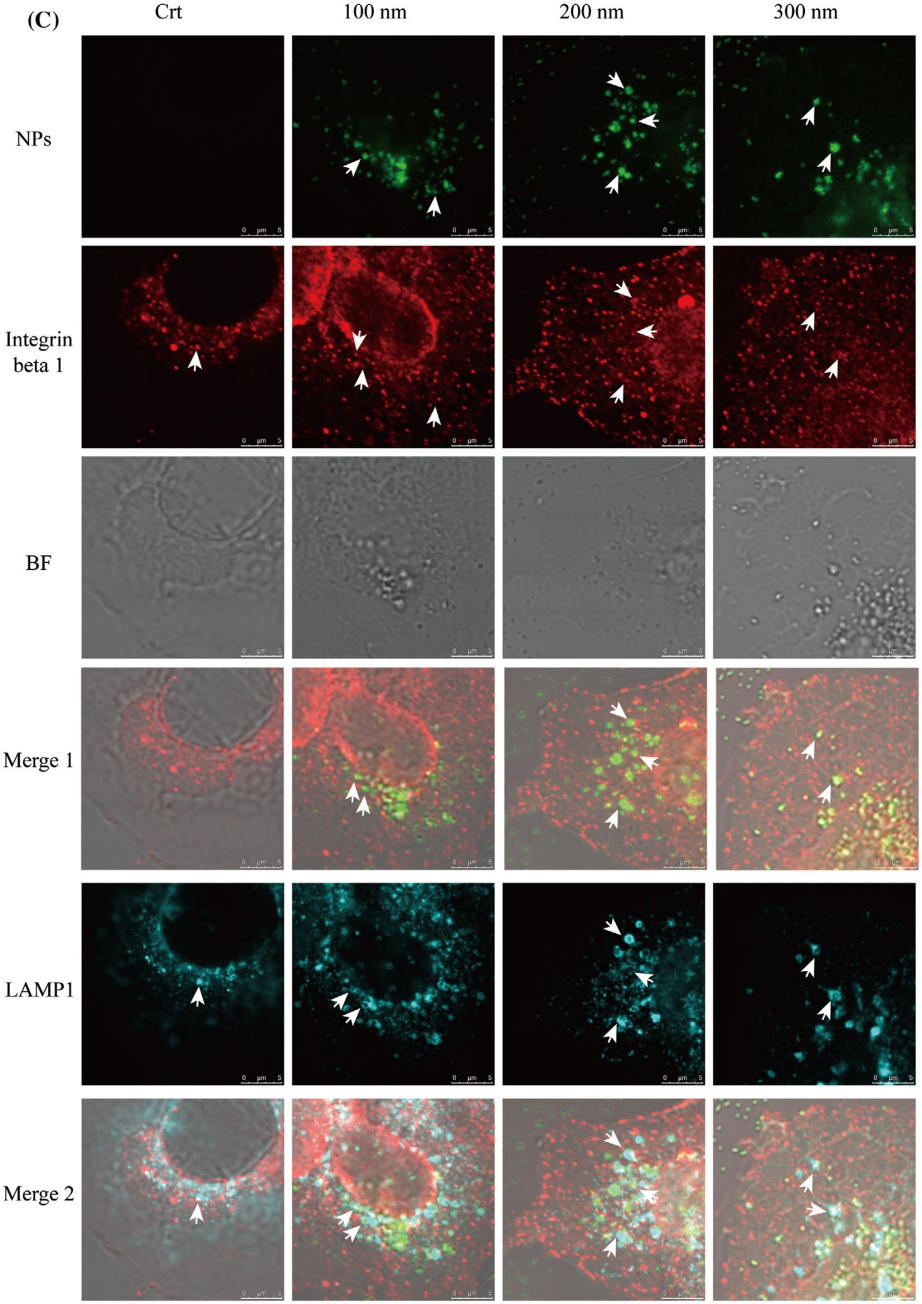

**Figure:**
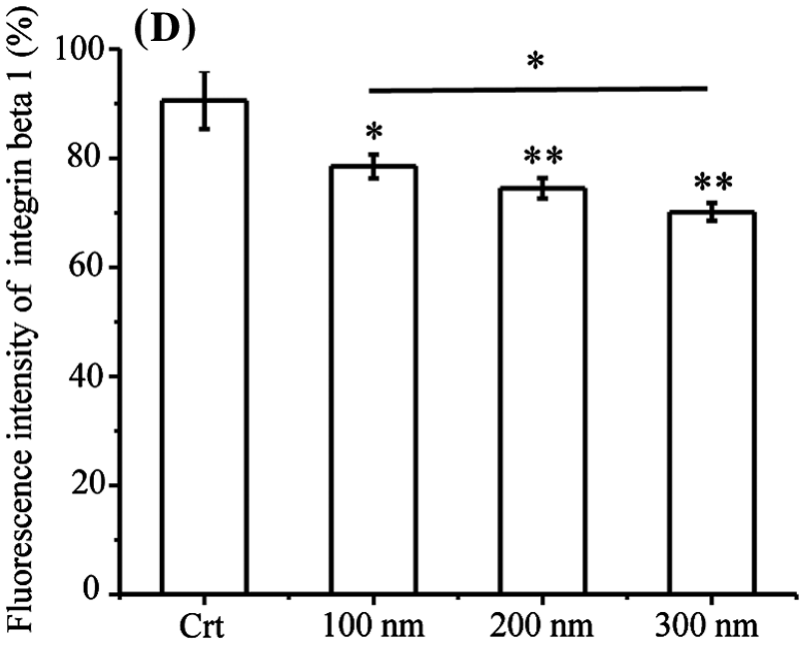

Focal adhesions play vital roles in cell migration, which requires endocytosis and recycling of integrins. Endocytic integrin beta 1 is sorted from the early endosome and directed to either late endosomes and lysosomes for degradation or recycling endosomes and the plasma membrane for assembly into new focal adhesions [39]. In addition, the trafficking of integrin beta 1 can be achieved through clathrin-dependent endocytosis and clathrin-independent endocytosis (caveolin-dependent endocytosis and macropinocytosis) [27], which are the endocytic pathways utilized by TiO_2_-PEG NPs [31]. NPs with the diameters of 100 and 300 nm NPs are initially internalized through clathrin-mediated endocytosis and macropinocytosis and then separately transferred to early endosomes and macropinosomes, finally being localized to the lysosomes for clearance or degradation. Two-hundred nanometer NPs are internalized through both clathrin-mediated endocytosis and micropinocytosis and transferred to early endosomes and macropinosomes and finally lysosomes. Early endosomes and macropinosomes can be marked using the EEA1 antibody [40] and lysosomes can be marked using the LAMP1 antibody. Therefore, to confirm the involvement of integrin beta 1 in the endocytosis of NPs, we tracked the localization of integrin beta 1, early endosome/macropinosome or lysosomes and NPs at 3 h. As shown in Figure 2(B), early endosomes and macropinosomes were labeled by the EEA1 antibody in blue, integrin beta 1 was labeled with the anti-integrin beta 1 antibody in red and TiO_2_-PEG NPs were labeled by FITC in green. Additionally, bright field (BF) images were obtained to visualize the localization of cell structures. Firstly, the role of NPs during the endocytic process of integrin beta 1 was studied. As shown in merge 1 (Figure 2(B)), integrin beta 1 was co-localized with NPs, as directed by the white arrows, which suggested the binding of NPs and integrin beta 1. Following that, more combined NPs with integrin beta 1 were found in the EEA1-positive endosomes compared with the control (merge 2, Figure 2(B)), indicating promoted endocytosis of integrin beta 1 by NPs. Thereafter, the lysosomes, integrin beta 1 and NPs were tracked at 3 h (Figure 2(C)). In the control group, the integrin beta 1 and lysosome loops were co-localized as indicated by the white arrows in the merged figure (merge 2, Figure 2(C)), indicating the occurrence of lysosomal degradation of integrin beta 1 under normal conditions. After incubation with NPs for 3 h, the co-localization of NPs with integrin beta 1 was also found in merge 1 (white arrows, Figure 2(C)), and the lysosome loops increased compared to control cells. Additionally, FITC-labeled NPs were encapsulated in two or three by the lysosomal loops, and integrin beta 1 was distributed on or inside the lysosomes loops with NPs, as indicated by the white arrows. With larger NPs, more integrin beta 1 receptors were encapsulated with NPs by the lysosomes loops. Therefore, the localization analysis in Figure 2(B) and (C) indicates that endocytosis of integrin beta 1 is promoted through the combining of NPs with integrin beta 1 and then translocated to lysosomes. Furthermore, expression of integrin beta 1 was significantly decreased after exposure to NPs (Figure 2(D)), compared with control cells. Moreover, the expression of integrin beta 1 following treatment with 300 nm NPs was lower than that of cells treated with 100 and 200 nm NPs, which suggests that the size of NPs affects endocytic pathways of NPs and integrin beta 1 and thereby integrin beta 1 expression. The results showed that the decreased expression of integrin beta 1 observed in the cytoplasm is probably caused by lysosomal degradation.

Therefore, the quantification and co-localization results suggest that the combining of NPs with integrin beta 1 promotes endocytosis of integrin beta 1 and translocation to lysosome, leading to decreased the expression of integrin beta 1.

**Figure 3.**
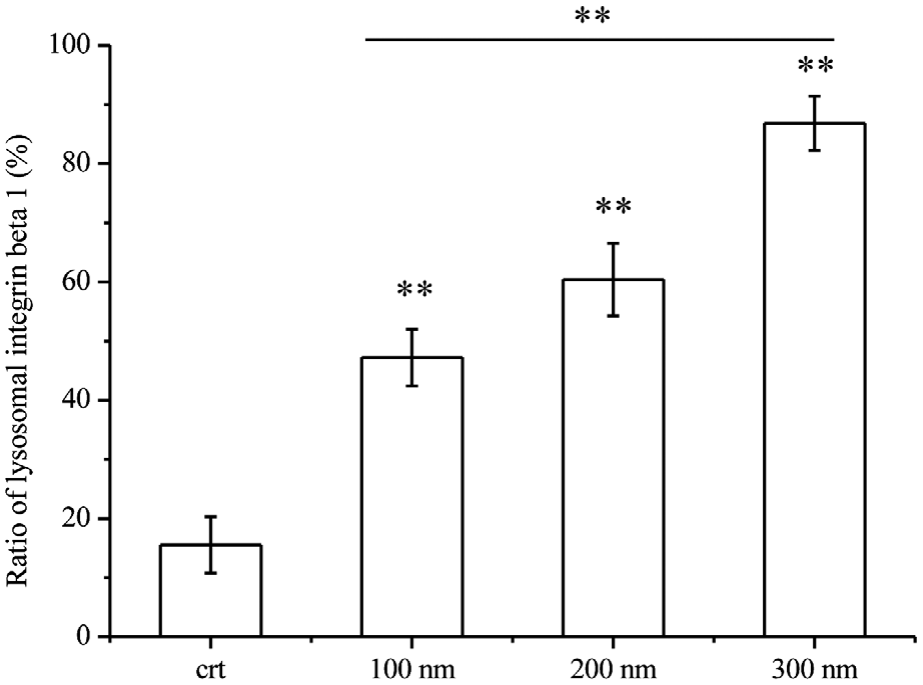
Ratios of lysosomal integrin beta 1 with or without NPs exposure. Cells were seeded and exposed to the TiO_2_-PEG NPs (100, 200, and 300 nm) at a concentration of 100 μg/mL for 3 h. After observation by confocal microscope, the numbers of lysosomes, lysosomal integrin beta 1, and lysosomal integrin beta 1 with NPs were counted and calculated as shown above. For each group, five cells were selected for counting and calculation. Crt stands for control samples. All data are displayed as mean ± SD with at least three parallel groups and were analyzed using one-way ANOVA. *p ≤ 0.05, **p ≤ 0.01.

In normal conditions, the balance between the lysosomal and recycling integrin beta 1 maintains the normal cell migration. When the balance of integrin beta 1 was damaged, the cell migration would be affected. Our work showed the decreased expression of integrin beta 1 with a size-dependent manner after treatment of NPs. Therefore, to confirm the effects of NPs on lysosomal integrin beta 1, the ratios of lysosomal integrin beta 1 were counted and displayed in Figure 3. The result showed that after NPs’ exposure, the lysosomal integrin beta 1 increased significantly compared with that of control. Besides that, larger NPs induced more integrin beta 1 to lysosomes. Pearson correction coefficient between NPs’ size and lysosomal integrin beta 1 was 0.98, showing a size-dependent mediation of lysosomal integrin beta 1. Therefore, based on above work, the results indicated that the decreased expression of integrin beta 1 was mediated by NPs, which misrouted more integrin beta 1 to lysosome and thereby decreased its expression.

### 3.3. Expression of pFAK is decreased after exposure to NPs

**Figure 4.**
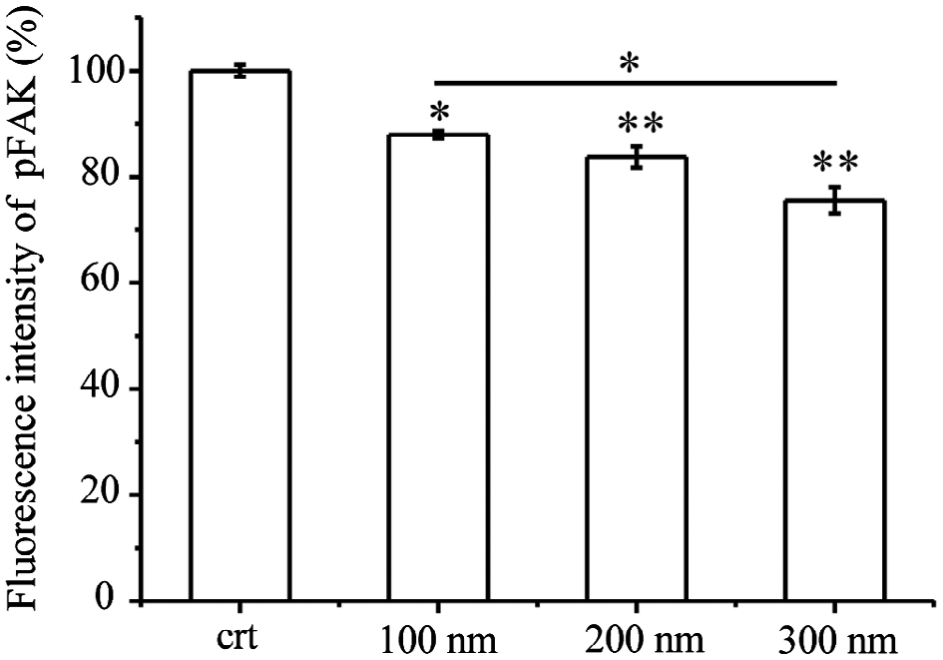
Quantification of pFAK. Cells were seeded and exposed to the TiO_2_-PEG or FITC-TiO_2_-PEG NPs (100, 200, and 300 nm) at a concentration of 100 μg/mL for 3 h. Crt stands for control samples. All data are displayed as mean ± SD with at least three parallel groups and were analyzed using one-way ANOVA. *p ≤ 0.05, **p ≤ 0.01.

FAK is a member of a family of non-receptor protein-tyrosine kinases that regulate integrin and growth factor signaling pathways involved in cell migration, proliferation, and survival [41]. In addition, FAK is one of the first downstream signaling components to become activated by integrins. Based on the observed decrease in integrin beta 1 expression, the expression of pFAK was determined using flow cytometry. As shown in Figure 4, pFAK expression was quantified after exposure of TiO_2_-PEG NPs to cells for 3 h. The results showed that pFAK expression was significantly decreased following exposure to 100–300 nm TiO_2_-PEG NPs.

### 3.4. F-actin structures are de-bundled and disrupted after NPs treatment

**Figure 5.**
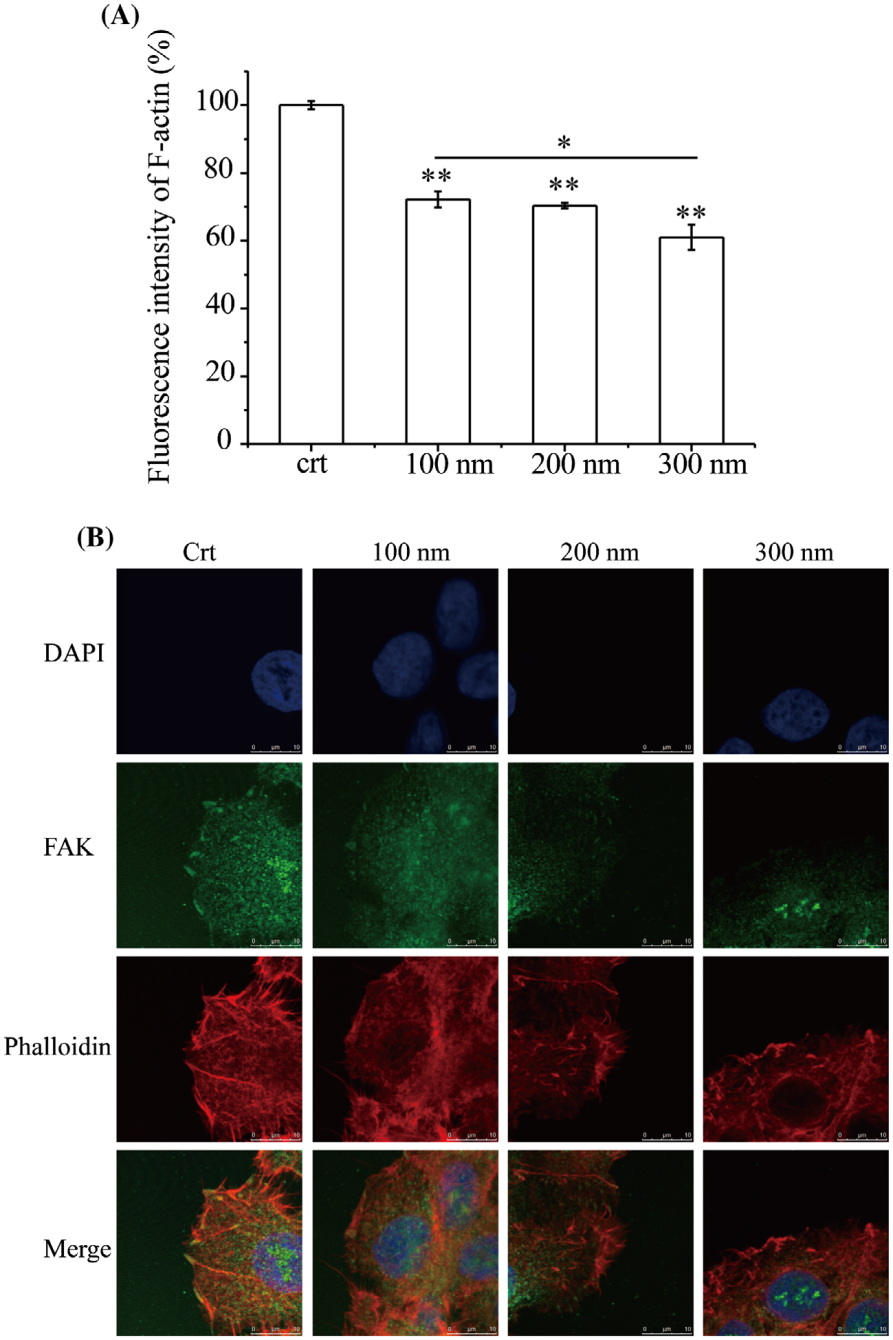
Expression and localization of F-actin. Cells were seeded and exposed to the TiO_2_-PEG NPs (100, 200, and 300 nm) at a concentration of 100 μg/mL for 3 h. (A) Quantification of f-actin expression; (B) Localization and structural assessment of f-actin and pFAK. Crt stands for control samples. All data are displayed as mean ± SD with at least three parallel groups and were analyzed using one-way ANOVA. *p ≤ 0.05, **p ≤ 0.01.

During migration, a cell first extends protrusions (lamellipodia and filopodia), forms adhesions, and finally retracts its tail. The actin cytoskeleton plays a major role in this process [42]. pFAK modulates F-actin dynamics, including actin cytoskeleton polymerization and lamellipodia protrusion, and loss of pFAK reduces the maintenance of the integrity of bundled actin filaments [43,44]. Based on the observed decrease in pFAK expression, F-actin expression after NP exposure for 3 h was assessed by flow cytometry (Figure 5(A)). Compared with control cells, F-actin expression was significantly decreased after exposure to NPs for 3 h. Again, the effect was proportional to the size of the NPs, with F-actin expression significantly decreased between the cells treated with 100 and 300 nm NPs. Furthermore, the localization of pFAK (green) and F-actin (red) was observed by confocal microscope (Figure 5(B)). In the control cells, pFAK was localized at the edge of the cell membrane and F-actin was bundled with lamellipodia and filopodia protrusions which related to pFAK. After treatment with NPs for 3 h, F-actin was de-bundled and disrupted, and cells lacked protrusions, indicating that cellular motility would be decreased. Consistent with previous observations, more F-actin structures were fragmented by larger NPs.

pFAK could sustain the stability of the lamellipodia protrusions of f-actin through combining with them. With the decreased expression of pFAK, the combining became unstable, leading to the disrupted f-action and adverse effect to cell migration. Therefore, the results suggest that the decreased pFAK expression de-bundled F-actin and disrupted the cellular cytoskeleton.

Our results showed that the expression of F-actin was significantly decreased and F-actin structures were de-bundled and disrupted after NP exposure, which accounted for the inhibition of cell migration.

## 4. Discussion

Integrin beta 1, as the major fibronectin receptor on most cells, transmits cellular signaling across cell membrane to modulate cell adhesion, survival, and migration. Its endocytic trafficking is divided into two parts based on the trafficking time: one is long loop trafficking during which integrin beta 1 is recycled to the plasma membrane for new focal adhesions formation and cell migration; the other one is short loop trafficking during which integrin beta 1 is sorted and translocated to the late endosome or lysosome for degradation [45]. The equilibrium between recycling and degradation of integrin beta 1 maintains the normal cell migration, which plays a crucial part in embryo development, histogenesis, and wound healing [46].

However, exposure of NPs struck the balance of integrin beta 1, as they share the same endocytic pathways but different exocytosis pathways [47,48]. NPs, after endocytosis into cytoplasm, partly remain in the cells for a long time, such as macrophage, or are transferred outside of cytoplasm through exocytosis, including lysosomes, multivesicular bodies, and other pathways [48]. In our work, following exposure of NPs to cells for 3 h, more integrin beta 1 was found firstly in EEA1-positive endosomes and then lysosomes compared with control (no NPs exposures to cells). Furthermore, insides of both endosomes, NPs were found to combine with integrin beta 1. These results indicate that NPs firstly combined with integrin beta 1 at the beginning of endocytosis, and then the combination of NPs and integrin beta 1 was internalized through corresponding pathways which were dependent on sizes of NPs. Therefore, NPs triggered and promoted the endocytosis of integrin beta 1. Following that, endocytic combination of NPs and integrin beta 1 was sorted and translocated to lysosome, which probably decreased the expression of integrin beta 1 through lysosomal degradation. This misrouting of integrin beta 1 to the degradation pathway (lysosomes) rather than recycling pathways reduced cell spreading on fibronectin [49]. However, the lysosomal integrin beta 1 could be promoted to recycling from late endosomes or lysosomes by Rab25 and CLIC3 to drive cancer progression [50]. Therefore, during the trafficking of integrin beta 1 with NPs, if such recycling proteins participant and promoted the cycling of integrin beta 1 to plasma membrane, it still needs more work to confirm. Furthermore, the overexpression and downregulation of integrin beta 1 should be carried on in our work to prove the effects of NPs and integrin beta 1 on cell migration. However, integrin beta 1 affects many cellular functions, not only cell migration, but also cell proliferation, cell adhesion, and immune response. Therefore, over- and down-expression of integrin beta 1 would not only alter the function of cell migration, which is not suitable to our work.

pFAK is important for cell migration, as it could integrate growth factor and integrin signals to promote cell migration [51], and expression of the protein-tyrosine phosphatase (PTEN) induces dephosphorylation of FAK and inhibition of cell migration [52]. Besides that, cell spreading and migration were enhanced after overexpression of FAK, compared with those of FAK-null cells [53,54]. While FAK deficient cells display a rounded morphology, increased formation of cell-substratum contact sites and migration defects [54]. pFAK, as the transduction signaling of cell migration, is activated by integrins. So, less expression of integrin beta 1 reduced the activation of pFAK, leading to weaker signaling of cell migration. Besides that, pFAK could maintain the stability of protrusions (lamellipodia and filopodia) of f-actin to modulate cell migration [43,44,55]. Therefore, when the expression of pFAK was decreased, the integrity of bundled actin filaments was also damaged, which deceased the polarity of cellular skeleton and thereby cell migration.

Here, we report the cytotoxicity of NPs from the integrin beta 1-mediated inhibition of cell migration. Larger NPs significantly decreased cell migration much stronger. The integrin beta 1-related inhibition of cell migration provides information on the cancer therapy through inhibiting cancer invasion by targeting integrin beta 1 to degradation routing.

## 5. Conclusions

**Figure 6.**
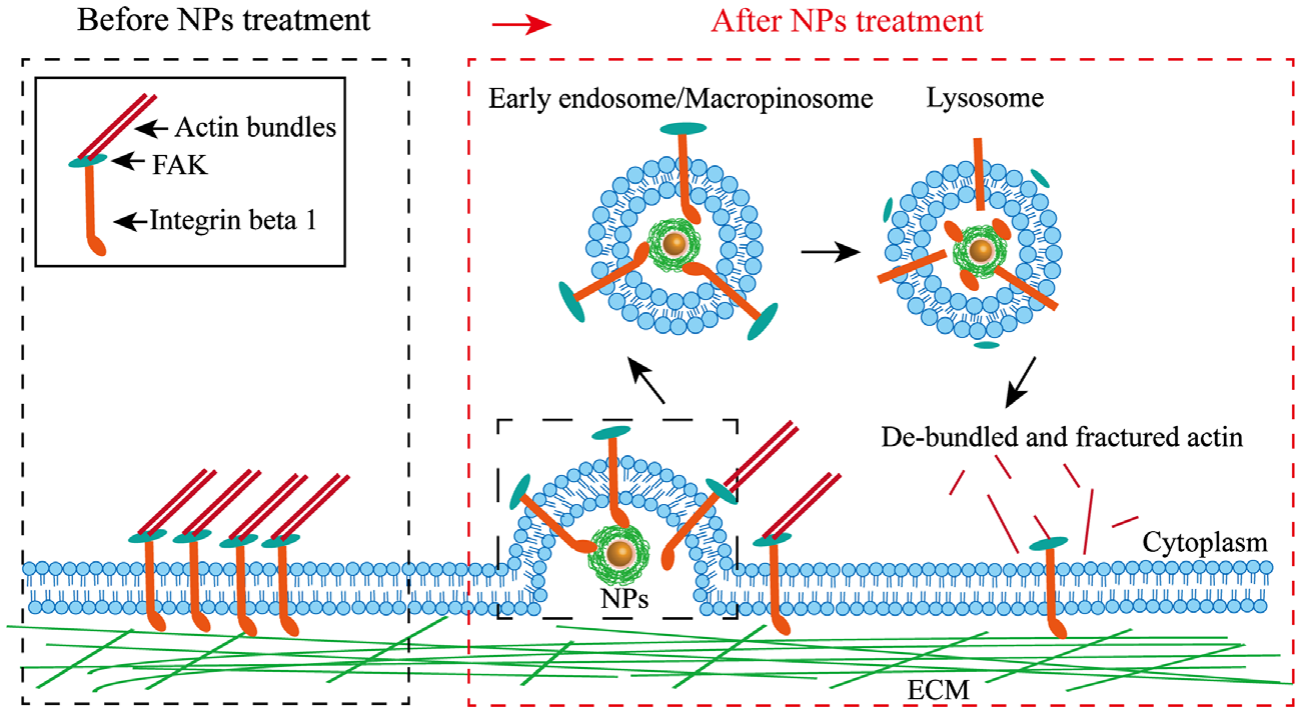
Schematic of the possible mechanisms of the inhibition of cell migration caused by NPs via integrin beta 1.

Migration of NCI-H292 cells was inhibited by TiO_2_-PEG NPs, which was mediated by integrin beta 1. The possible mechanism of integrin-mediated inhibition of cell migration is displayed in Figure 6. The combination of NPs with integrin beta 1 promoted the endocytosis of integrin beta 1 from EEA 1-positive endosomes to lysosomes, which decreased the expression of integrin beta 1. Following that, pFAK expression was decreased due to the reduced activation from upstream integrin beta 1, which weakened the binding between pFAK and f-actin and led to de-bundled and disrupted f-actin structure, thereafter inhibiting cell migration. Furthermore, size of NPs played an important role in cellular uptake pathways and ratios. The larger NPs in our work possessed higher cellular uptake ratios, which significantly inhibited cell migration through decreasing the expression of integrin beta 1, pFAK, and f-actin. Therefore, cell migration depends on not only surface receptor, but also NPs sizes, which provides valuable information for cancer therapy through NPs-mediated integrin beta 1.

## Disclosure statement

No potential conflict of interest was reported by the authors.

## Funding

This work was supported by the National Institute for Materials Science.

## Supplemental data

Supplemental data for this article can be accessed at https://doi.org/10.1080/14686996.2018.1444318.

## Supplementary Material

Supplementary.zip
