# Supplementary figures and images for: PEGylated TiO2 nanoparticles mediated inhibition of cell migration via integrin beta 1
Source: Sci Technol Adv Mater. 2018 Mar 8;19(1):271–81. doi: 10.1080/14686996.2018.1444318 (PMC5917434; doi:10.1080/14686996.2018.1444318)

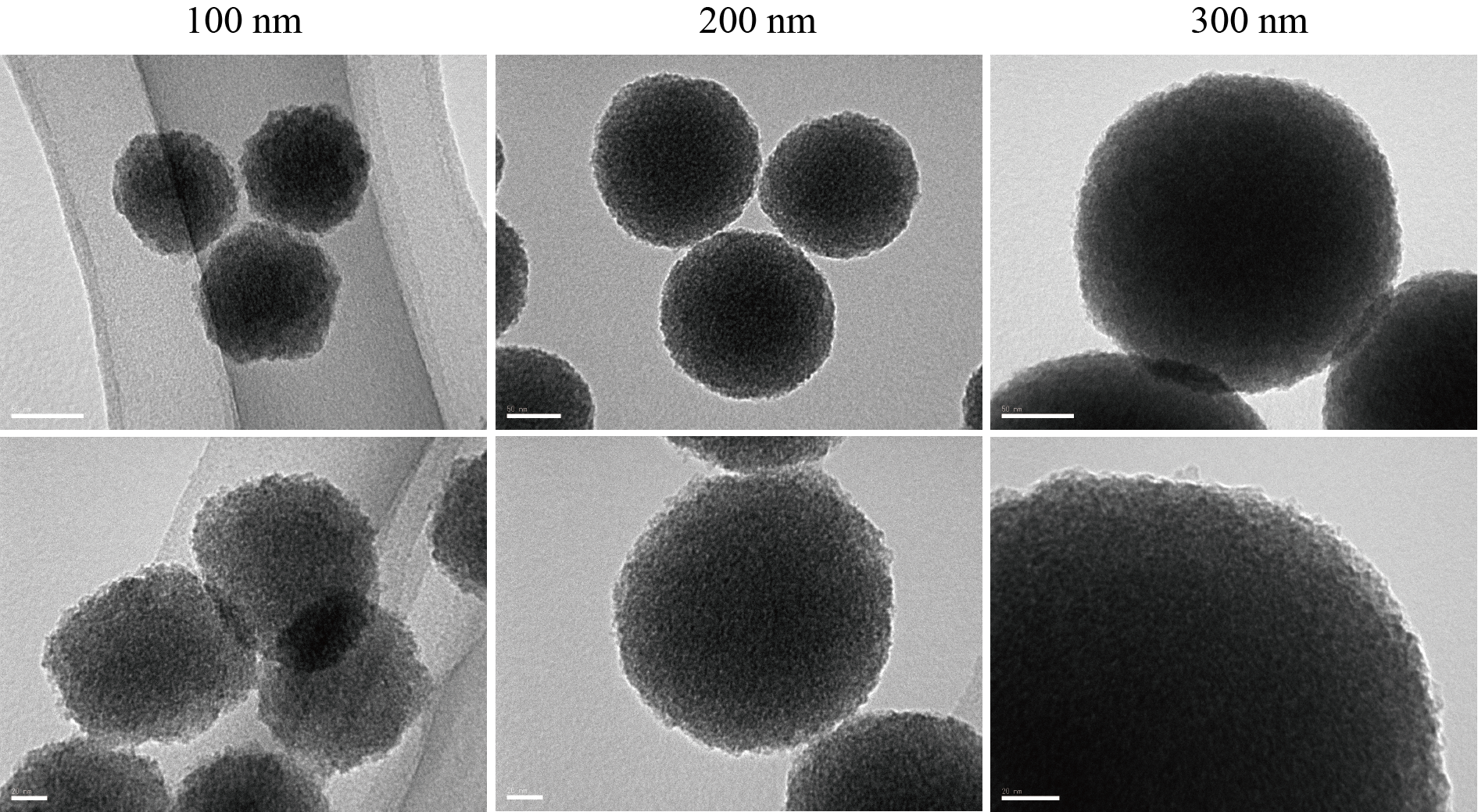

Supplement: Supplementary.zip [file TSTA_A_1444318_SM6687.zip › Figure S1.png]

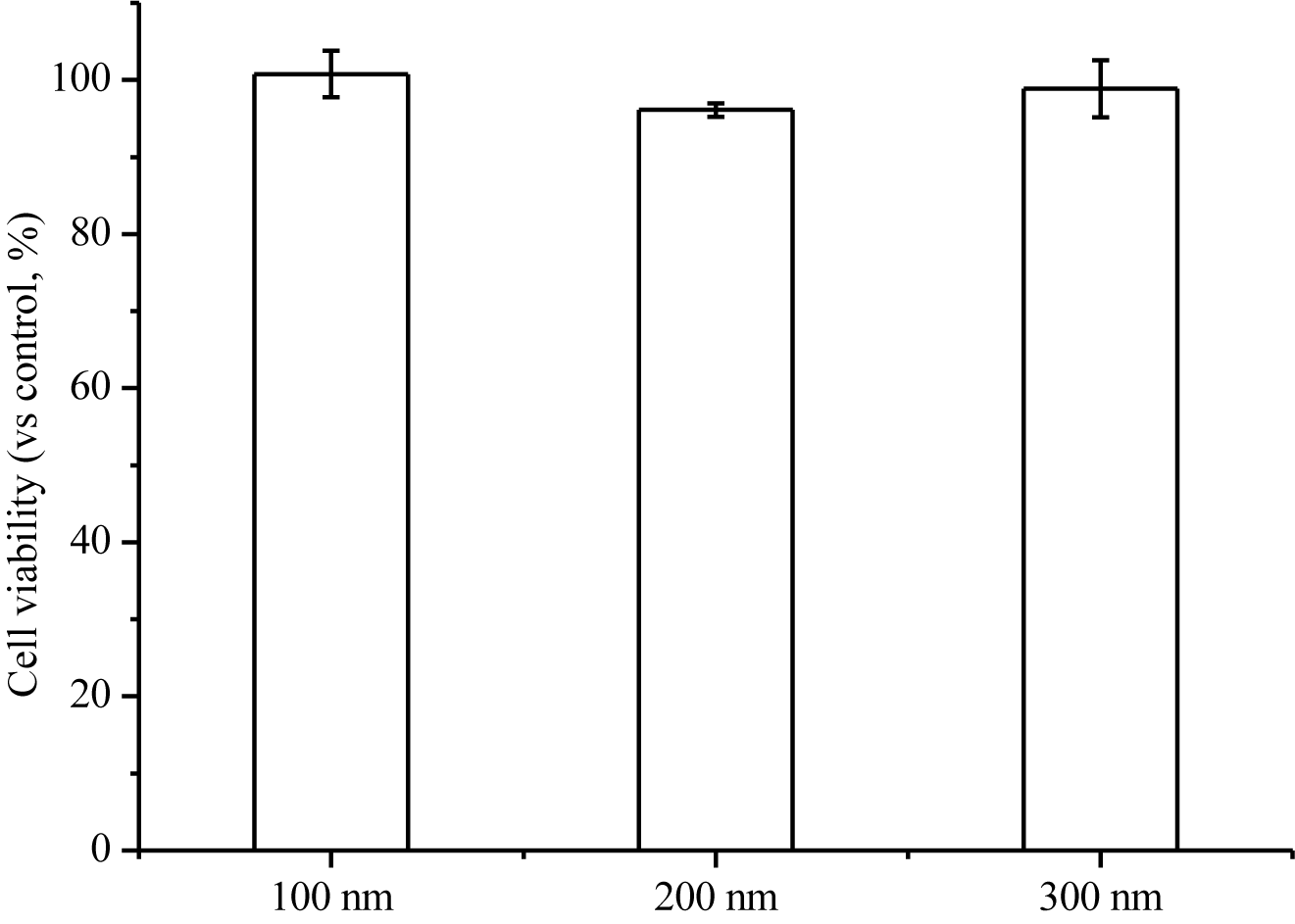

Supplement: Supplementary.zip [file TSTA_A_1444318_SM6687.zip › Figure S2.png]
